# Cyclic Peptide–Polymer Conjugate Nanotubes for Delivery of SN‐38 in Treatment of Colorectal Cancer Model

**DOI:** 10.1002/adhm.202502527

**Published:** 2025-10-10

**Authors:** Sophie K. Hill, Min Zeng, Santhosh Kalash Rajendrakumar, Robert Dallmann, Sébastien Perrier

**Affiliations:** ^1^ Department of Chemistry University of Warwick Gibbet Hill Road Coventry CV4 7AL UK; ^2^ Warwick Medical School University of Warwick Coventry CV4 7AL UK; ^3^ Zeeman Institute for Systems Biology and Infectious Disease Epidemiology Research University of Warwick Coventry CV4 7AL UK; ^4^ Faculty of Pharmacy and Pharmaceutical Sciences Monash University 381 Royal Parade Parkville VIC 3052 Australia

**Keywords:** cancer therapy, colorectal cancer, cyclic peptide, drug delivery, peptide–polymer conjugates

## Abstract

Cyclic peptide‐polymer conjugate nanotubes have been shown to be powerful drug delivery vectors, due to their propensity for dynamic self‐assembly, high aspect ratio morphology and structural interchangeability. Building upon previous studies that demonstrate the shielding abilities of the polymeric corona of nanotubes to enhance pro‐drug bond stabilities and modulate hydrolysis, here the concept of a hydrophobic core building block with multiple drug units to improve drug loading capacity and overall efficiency of the nanotube carriers is utilized. By leveraging the intermolecular features of the drug core to strengthen assembly, it is hypothesized that these nanotubes have the potential as a responsive supramolecular delivery system whereby upon full hydrolysis of the labile drug, these core forming interactions disappear, and nanotubes can fall apart and undergo clearance. Herein, the self‐assembly, in vitro efficacy and in vivo pharmacokinetic and anti‐tumor pharmacodynamics of these nanotubes in colorectal cancer models, comparing the potent topoisomerase inhibitor SN‐38 with its clinically‐used parent pro‐drug irinotecan, is explored.

## Graphical abstract

This study develops cyclic peptidepolymer conjugate nanotubes with hydrophobic, drug‐rich cores to enhance drug loading and stability, and demonstrates their responsive disassembly, pharmacokinetics, and anti‐tumour efficacy in colorectal cancer models using the anticancer drug SN‐38.

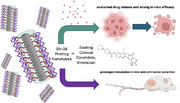

## 1 Introduction

Despite significant scientific advances in recent decades, cancer remains one of the most formidable challenges for modern medicine. The rising rates of colorectal cancer (CRC) diagnoses, particularly amongst younger people, exemplify the new complexities faced. Despite efforts to promote early detection and fast onset of treatment, the prognosis for CRC remains grim, ranking as one of the deadliest malignancies worldwide. In 2020 alone, there were an estimated 1.9 million new cases and 930 000 deaths attributed to colorectal cancer, with projections estimating 3.2 million new cases annually within the next two decades.^[^ ^1^ ^]^ Current strategies for early‐stage CRC typically rely on surgical resection, paired with radiotherapy or adjuvant chemotherapy utilizing potent cytotoxic drugs to target remaining cancerous tissues. However, these drug agents exhibit low efficacy and are accompanied by significant debilitating side effects due to their lack of specificity, causing damage to healthy tissues and requiring extremely high doses to achieve therapeutic impact due to rapid clearance. Efforts to optimize topoisomerase inhibitors such as the camptothecins have resulted in highly potent drugs such as SN‐38 (7‐ethyl‐10‐hydroxycamptothecin), which demonstrate broad‐spectrum activity at nanomolar concentrations against various cancer types. However, these properties are hindered by poor solubility, adverse effects, and rapid clearance.^[^ ^2^ ^]^ Consequently, the more hydrophilic and longer‐circulating prodrug, irinotecan, emerged as a typical chemotherapeutic in the clinical setting.^[^ ^3^ ^]^ Nevertheless, its potency is reduced 100–1000 fold compared to SN‐38 due to significant variability in the rate of enzymatic prodrug metabolism (typically <10%), and it still incurs serious side effects including myelosuppression and life‐threatening gastrointestinal toxicities.^[^ ^4^ ^]^ Efforts have naturally gravitated toward bypassing issues associated with the necessity for high doses of irinotecan, by directly manipulating native SN‐38 to target its potency to the desired site at a sustained therapeutic concentration without off‐target effects. One approach is through the use of nanoscale drug delivery vehicles, which can achieve more favorable biodistribution profiles from extended circulation times, affording preferential accumulation in tumor tissues as a result of the passive enhanced permeability and retention (EPR) effect.^[^ ^5^ ^]^ To date, a range of materials, including polymer conjugates, polymer nanoparticles, polymer micelles, liposomes, and antibody‐drug conjugates have shown promise as strong candidate excipients that improve experimental outcomes in preclinical anti‐tumor studies.^[^ ^6^, ^7^ ^]^ For example, England et al. reported complete regression of SW620 human colon cancer xenografts and minimal gastrointestinal toxicity in a mouse model, highlighting the efficacy of their SN‐38 prodrug polyoxazoline based dendrimers.^[^ ^8^ ^]^ Additionally, supramolecular delivery systems have gained exposure in recent years as a tool by which to manipulate not just particle size and morphology, but also stability and release of drug cargo for precise tuning of formulation activity over time, and in specific biological environments.^[^ ^9^ ^]^ Additionally, the ability to utilize drugs as key driving forces of particle self‐assembly allows for the creation of well‐defined structures, often accompanied by a high wt.% drug content, and tunable release which is readily controllable through a wide range of responsive prodrug linker chemistries.^[^ ^10^ ^]^

Previously, we determined that careful selection of prodrug linker type alongside polymer architecture for modifying camptothecin‐loaded cyclic peptide‐polymer conjugate nanotubes can afford sustained drug release profiles with significant anti‐proliferative activity against both 2D and 3D in vitro models for multiple cancer cell lines.^[^ ^11^ ^]^ Moreover, the nanotube supramolecular design based on extended alternating D/L cyclic peptide beta‐sheet networks to afford high aspect ratio tubes is beneficial for extended circulation times, improved penetration through tumor tissues, and eventual clearance upon disassembly of these reversible building blocks.^[^ ^12^ ^]^ For example, Kerr et al. reported the advantageous ratio of circulation time versus renal clearance in comparison to linear polyethylene glycol, or covalent polymer brushes.^[^ ^13^ ^]^ Additionally, Rho et al. established that incorporation of amphiphilic polymers around the cyclic peptide core leads to the stabilization and increased size of nanotubes ^[^ ^14^ ^]^ which was later determined to have an additive shielding effect on prodrug stability for drugs tethered to the peptide.^[^ ^11^ ^]^ We hypothesized that replacing the hydrophobic monomer in the inner diblock polymer shell, with multiple SN‐38 units, could instruct intermolecular forces (hydrophobic barrier, *π−‐π*) to strengthen nanotube assembly and facilitate disassembly upon prodrug ester hydrolysis and drug delivery. Moreover, compared to previous camptothecin materials, which exhibited one drug per peptide, this new design could dramatically improve the payload per conjugate for a more efficient use of the carrier materials. In this work, we synthesize the proposed SN‐38 peptide‐polymer conjugates and investigate their self‐assembly properties, alongside the in vitro and in vivo performance in a colorectal cancer model, comparing to the free drug, or clinical candidate irinotecan to assess the potential for these novel delivery vectors as an alternate therapeutic avenue.

## 2 Results and Discussion

### 2.1 Synthesis

**Figure 1:**
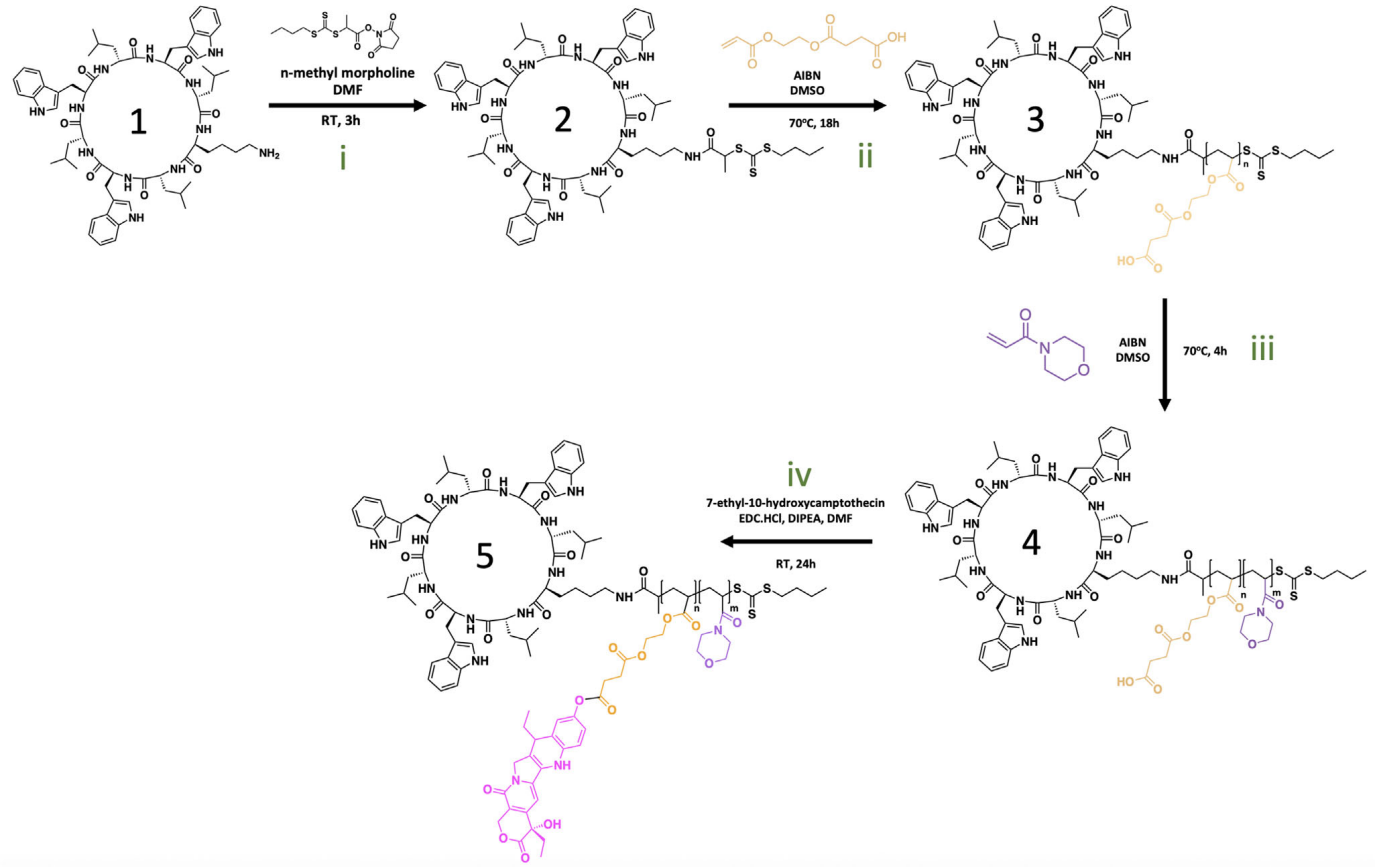
Synthetic protocol schematic for the multi‐step synthesis of SN‐38‐loaded peptide‐polymer conjugates. i) installation of RAFT CTA onto peptide, ii)“grafting from” RAFT polymerization of HEA‐SA monomer, iii) “grafting from” chain extension with NAM, iv) drug‐loading of SN‐38 onto the polymer via esterification. (n = 10 HEA SA units, m = 80 NAM units).

**Figure 2:**
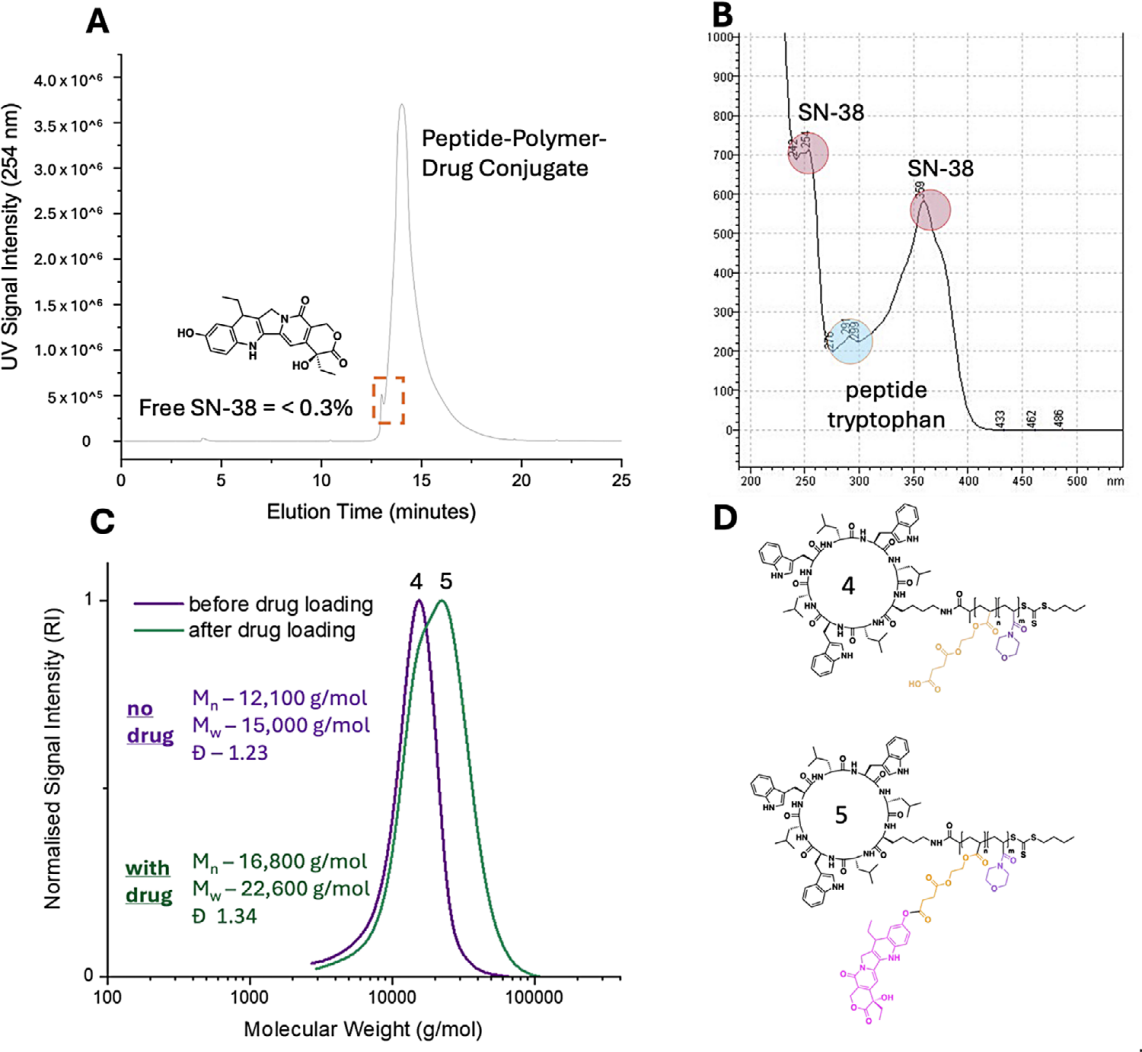
A) HPLC‐UV Chromatogram of drug‐peptide conjugate post purification (free drug quantified as < 0.3% of sample), B) UV absorbance spectrum of purified conjugate with distinct peaks for peptide (290 nm – tryptophan) and SN‐38 (245 nm, 360 nm), C) GPC‐SEC (DMF) Chromatogram of peptide‐polymer conjugate pre‐and post‐drug loading. D) Chemical structures of the drug‐free and drug‐conjugated peptide‐polymer species.

The synthetic method for generating drug‐loaded‐peptide polymer conjugates with a specific internal block available for drug loading proceeded via the “grafting” from RAFT polymerization method,^[^ ^15^, ^16^ ^]^ in which a diblock copolymer (with one carboxylic acid functionalized monomer for drug conjugation) was grown directly from the peptide itself. To achieve this, the cyclic peptide (previously optimized and synthesized by SPPS, see SI) was modified with a RAFT chain transfer agent (CTA), PABTC, via amide coupling between the pendant lysine and a NHS‐modified CTA, in the presence of a base (**Figure** **1**i). The reaction achieved complete conversion as demonstrated by ESI‐MS, HPLC, and NMR characterization of the purified product (See SI). RAFT polymerization was undertaken in DMSO, by first dissolving the peptide at 70 °C, followed by the addition of monomer and initiator and degassing with N_2_ to remove oxygen (Figure 1‐ii,iii). Monomer concentration was kept low due to the low solubility of the peptide, and reaction times were therefore kept relatively long, 18 h for the first block (HEA‐SA) and 6 h for the second block (NAM), achieving > 99%+ monomer conversion in each case. The degree of polymerization for each block was 10 and 80, respectively. SEC‐GPC analysis confirmed the synthesis of well‐controlled peptide‐polymer conjugates of molecular weight correlating well with theory, and low dispersity. The conjugation of SN‐38 to the carboxylic acid of the inner polymer segment was achieved through EDC, via esterification coupling with excess reagents occurring preferentially at the phenol alcohol site (Figure 1iv). The reaction was worked up by numerous precipitations in ice‐cold methanol to afford the final product. HPLC‐UV confirmed the formation of one major drug‐loaded peptide‐polymer conjugate species, with specific UV absorbances for peptide tryptophan, SN‐38, and the successful purification of excess free drug, which comprised less than 0.3% of the sample (**Figure** **2**A,B). A comparison of the GPC before and after drug conjugation showed a molecular weight shift, which confirms the addition of multiple drug molecules to the conjugate. A slightly broader molecular weight distribution with a small shoulder at lower molecular weight was observed (Figure 2C), presumably due to the altered hydrodynamic radius of each polymeric coil upon significant loading of the hydrophobic/aromatic drug. Examination of the ^1^H proton NMR for the final species confirmed the loading of multiple drug units, with a forced hydrolysis experiment showing a 14 wt.% drug content, corresponding to an average of 6–7 drugs per conjugate, out of 10 possible sites. This 60–70% loading efficiency is attributed to the steric hindrance in conjugating the full 10 drugs in close proximity within the core forming block. This new synthetic protocol showed a significant improvement upon previous generations of peptide‐polymer‐drug conjugate materials, which exhibited drug loading of ≈3 wt. % or lower.^[^ ^11^ ^]^

### 2.2 Characterization of Self‐Assembly

**Figure 3:**
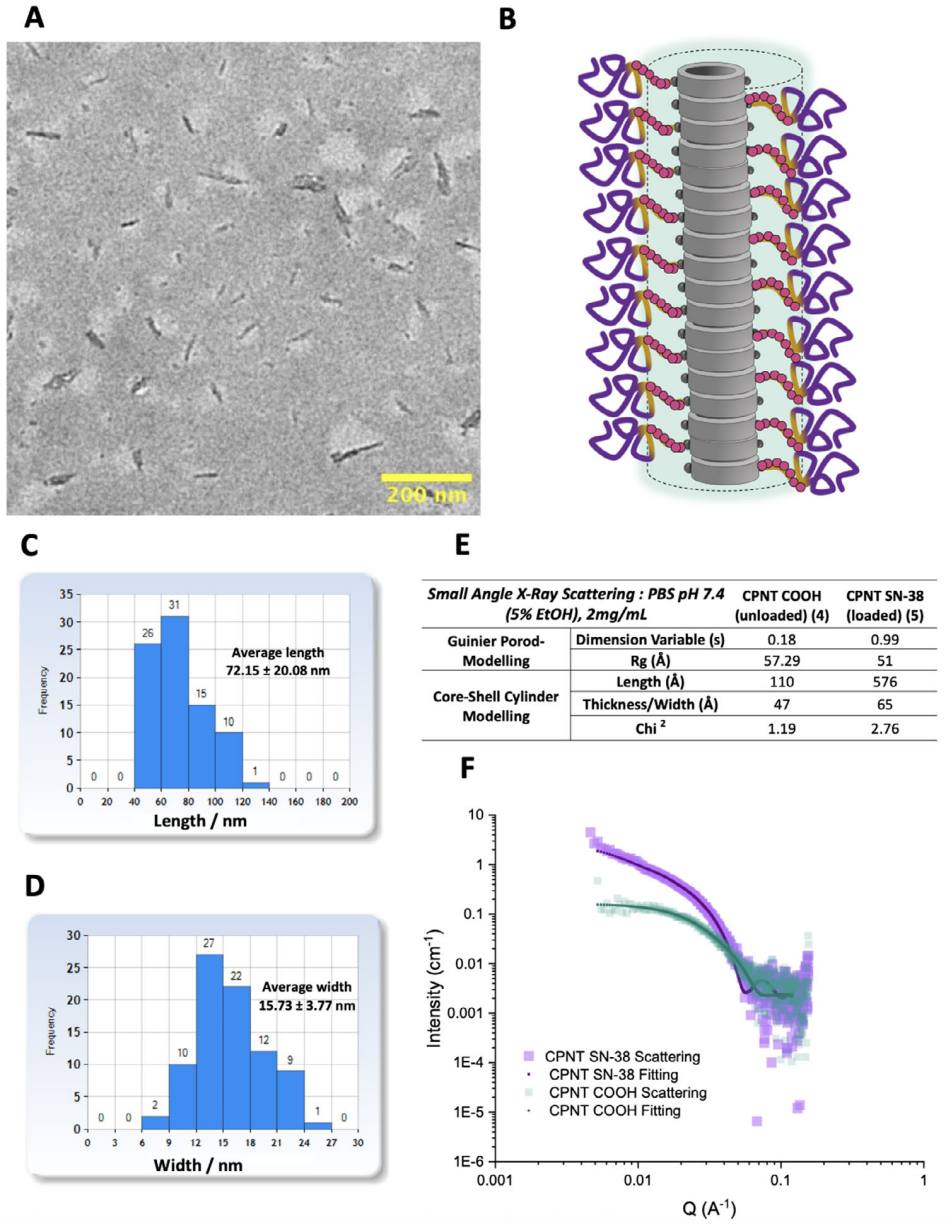
A) TEM image of assembled nanotubes. B) Illustration depicting the assembled drug loaded nanotubes. C+D) Distribution profiles of length and width for the assembled nanotubes measured from TEM images. E) Calculated SAXS parameters describing morphology/properties of nanotubes with and without drug when assembled in PBS (pH 7.4) and fit to specific models. F) SAXS scattering profiles of loaded/unloaded nanotubes alongside fitting profiles for core‐shell cylinder model.

Subsequently, the nanotubes were formulated in PBS (pH 7.4), and their self‐assembly properties explored by Small Angle X‐Ray Scattering (SAXS). By fitting to a simple Guinier‐Porod model, the scattering profiles shows how drug loading leads to elongation of nanotubes from short, almost globular structures, to distinct high aspect ratio nanomaterials, as indicated by the shift of dimension variable (s) from 0.18 to almost 1 (**Figure** **3**D). This elongation was confirmed in the core‐shell cylinder model which calculated lengths of 11 and 58 nm for unloaded/loaded nanotubes respectively (Figure 3D,E). Such a difference may be beneficial for a drug delivery system where cleavage of the drug at the target site leads to loss of drug‐drug stabilizing interactions, and electrostatic repulsion from carboxylate moieties, thereby instructing disassembly of the nanoparticle carrier and facilitating rapid clearance from the body. The efficiency of utilizing intrinsic drug features as key architectural blocks of supramolecular delivery systems has been increasingly emphasized in recent years.^[^ ^17^, ^18^, ^19^ ^]^ The nanotube morphology was further clarified by transmission electron microscopy (TEM), which provided images of the assembled drug loaded nanotubes, achieving average length and diameter of 72 and 15 nm (Figure 3A–C), in line with SAXS data.

### 2.3 In Vitro Efficacy

#### 2.3.1 Drug Release Profile

**Figure 4:**
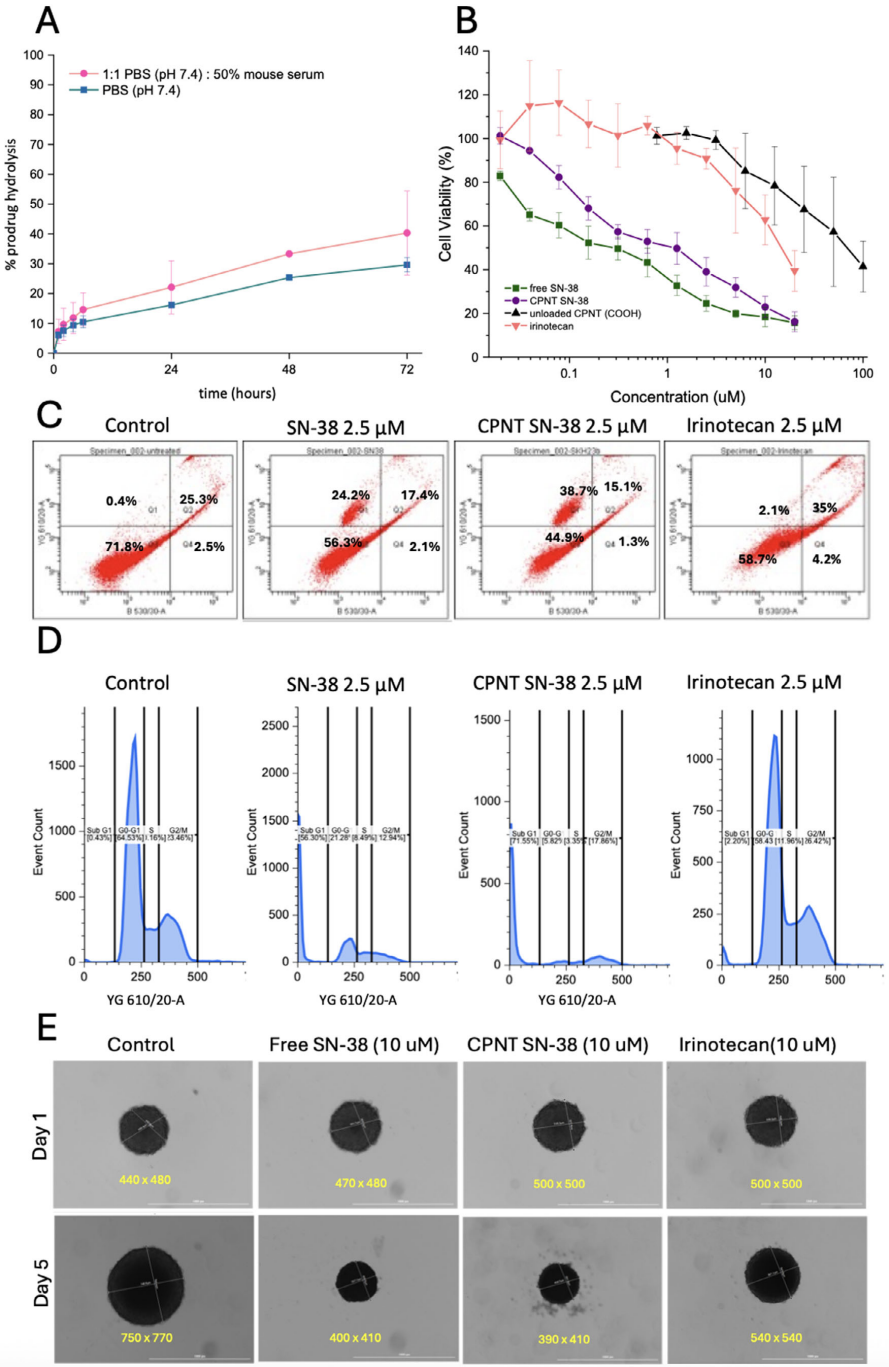
A) Profile of SN‐38 drug release from the nanotube carriers over 72 h in PBS (pH 7.4) or 1:1 PBS (pH7.4) and mouse serum. Error bars represent the standard deviation of the mean from 3 biological replicates (n = 3). B) Cell Viability (MTS Assay) for free SN‐38 drug, nanotube SN‐38 prodrug, irinotecan, or blank conjugates (COOH terminated) upon treating CT26 cells for 48 h. (note, concentrations are for SN‐38 concentration). Error bars represent the standard deviation of the mean from 3 biological replicates (n = 3). C) Flow cytometry quadrant profiles with PI and YO‐PRO staining for cells treated with 2.5 µM of drug for 48 h. D. Cell Cycle analysis and gating profiles with PI/RNase staining following treatment of CT26 cells for 48 h with 2.5 µM drug. E) Microscopy images of CT26 Spheroids grown and treated with 10 µM of drug for 5 days.

The nanotubes were prepared in PBS alone (pH 7.4) and in PBS supplemented with 50% mouse serum, respectively, and samples were analyzed by HPLC‐UV over time, to assess the drug release kinetics (**Figure** **4**A). Interestingly, the results revealed that the degree of hydrolysis after 72 h was just over 20% and 30% for the PBS and PBS + serum samples, respectively, which was faster compared to the previous generation of materials based on a hydrophobic butyl acrylate‐based core with the drug directly attached to the peptide.^[^ ^11^ ^]^ We postulate that the primary mechanism driving drug release is ester bond hydrolysis by water, with enzymatic activity – particularly from serum esterases – providing an additive effect that accelerates degradation in the presence of mouse serum. The faster release observed here compared to previous generations of materials can be explained by the difference in core design. In earlier systems, a single drug was attached to the peptide and surrounded by a hydrophobic polymer core that did not break down, maintaining a barrier that limited access to the drug at the peptide interface. In contrast, in the current system, the hydrophobic core is made up of the drug itself. As the drug undergoes hydrolysis, the inner shell becomes less hydrophobic, which in turn accelerates further hydrolysis. This faster breakdown of the hydrophobic barrier exposes charged carboxylate groups, leading to electrostatic repulsion that promotes nanotube disassembly and speeds up hydrolysis of the remaining cargo. Additionally, we propose that this process contributes to the supramolecular disassembly of the nanotubes into unimeric components in vivo, allowing eventual clearance from the body via renal filtration. Overall, the release profiles suggest sustained drug release over a prolonged duration, without burst release, which is favorable for achieving optimal therapeutic drug concentrations for in vitro and in vivo treatments.

#### 2.3.2 In Vitro Cell Viability

The in vitro performance of SN‐38 loaded nanotubes was evaluated in the murine CT26 colorectal cancer cell line, and compared to free drug and irinotecan, the clinically employed small molecule prodrug for SN‐38 used in the treatment of colorectal cancer. Treatment of cells and subsequent analysis of metabolic activity by MTS assay revealed that after 48 h, the free SN‐38 exhibited significant toxicity at low nanomolar concentrations (IC_50_ ≈200 nM), as expected (Figure 4B).^[^ ^20^, ^21^ ^]^ The drug‐free nanotubes showed some degree of toxicity, with concentrations near 100 µM required to achieve more than 50% cell death. We have observed some degree of toxicity for nanotubes in previous work, which we attribute to the nanotube's ability to disrupt the cell membrane.^[^ ^11^ ^]^ However, these concentrations far exceed what would be used in any realistic therapeutic context, and this is therefore not an issue for the development of these systems as drug delivery vectors. On the other hand, the nanotubes carrying SN‐38 were able to induce significant cell death in the nanomolar range (IC_50_ ≈500 nM), confirming successful drug release in the cellular environment, and posing as realistic therapeutic systems. Interestingly, irinotecan was markedly less potent, requiring concentrations over 10 µM to achieve more than 50% cell death. These data highlight the potential of nanotubes to replace this treatment, by introducing a highly potent drug such as SN‐38 in a more controlled and targeted manner via the carrier, potentially leading to fewer side effects in vivo. Additionally, CT26 cells were treated with each compound at 2.5 µM for 48 h and assessed for changes in apoptotic‐like features, and cell cycle distribution to compare the relative cytotoxic effects of the formulations. Flow cytometry staining with PI‐YO‐PRO (Figure 4C) showed that cells treated with free SN‐38 or SN‐38‐loaded nanotubes exhibited 45.7% and 55.1% total cell death (including early and late apoptotic populations), respectively, compared to 41.7% in irinotecan‐treated cells and 28.2% in untreated controls. Cell cycle analysis using PI/RNase staining (Figure 4D) revealed a marked increase in the sub‐G1 population for both free SN‐38 and the nanotube SN‐38 treatment groups, indicating elevated DNA fragmentation. This is in accordance with the known mechanistic activity of both drugs as topoisomerase I inhibitors, which results in DNA damage. These findings demonstrate that the loaded nanotubes exhibit a cytotoxicity profile favorably comparable to free SN‐38, whilst offering a clear improvement over the clinically used irinotecan prodrug. Furthermore, to assess the ability of different treatments to penetrate and induce cytotoxicity in more complex tumor models, 3D CT26 spheroids were generated and treated with free SN‐38 (10 µM), nanotube‐loaded SN‐38 (10 µM), or irinotecan (10 µM) for 5 days. Microscope images (Figure 4E) revealed distinct responses to each treatment. While control spheroids grew rapidly in size by over 300 µm in average diameter, irinotecan had a modest impact and was able to retard the rate of growth to just 40 µm increased diameter in the same timeframe. Interestingly, the free SN‐38, and remarkably the nanotube SN‐38 not only managed to completely inhibit growth, but also slightly reduce the average diameter, possibly due to a collapse in spheroid integrity.

These preliminary findings suggest that the SN‐38 prodrug nanotubes have strong potential as a supramolecular drug delivery system, which can offer similar potency to the free drug, improved efficacy over the clinical candidate irinotecan, and potentially have a more controlled release profile.

### 2.4 In Vivo Evaluation

#### 2.4.1 Biodistribution and Pharmacokinetics

**Figure 5:**
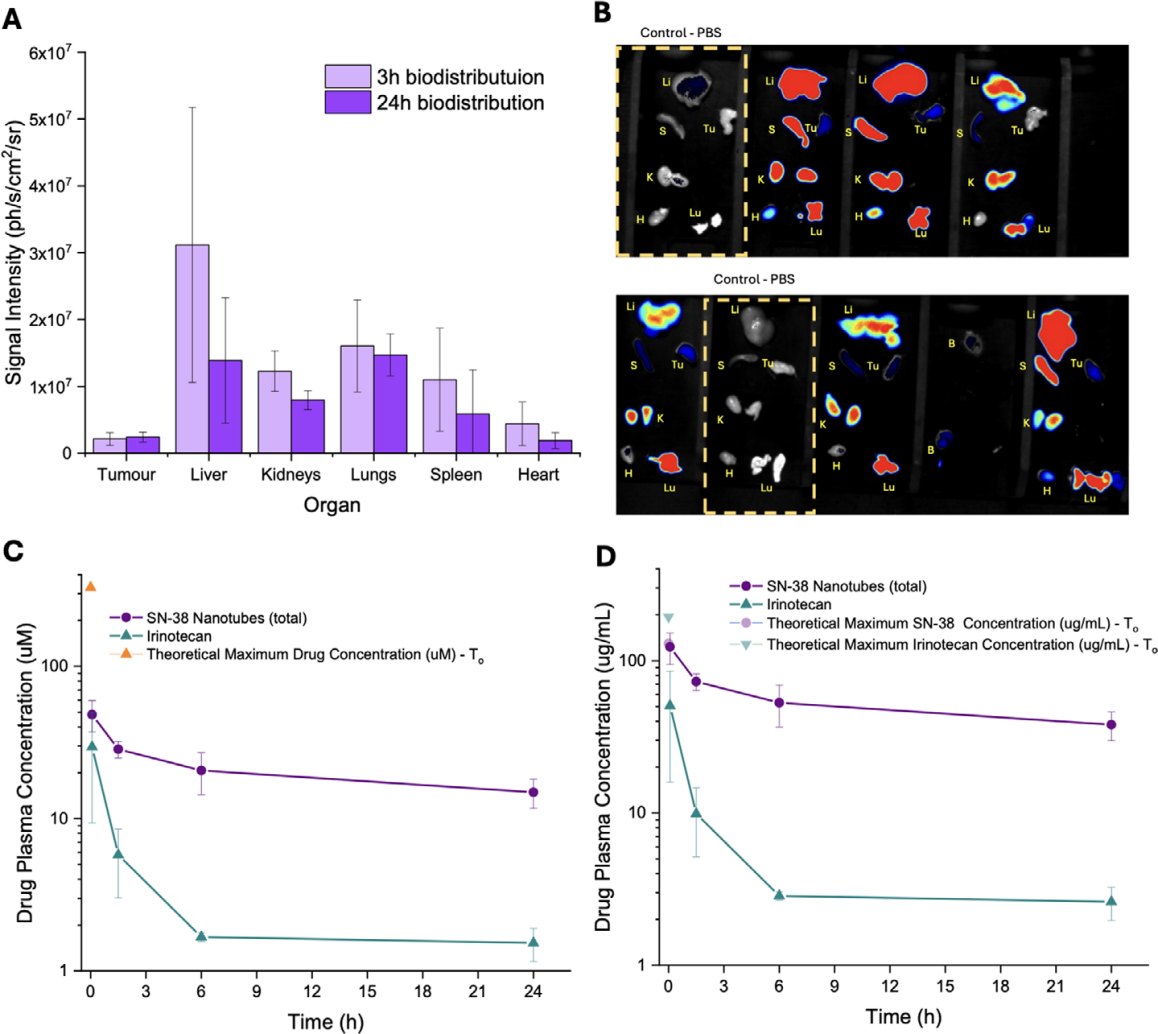
A) Biodistribution (ex vivo) results from measured signal intensity of NIR fluorophore labelled nanotubes in each organ 3 h/24 h post i.v injection in mice (n = 3). B) Actual images of ex vivo organs underneath optical NIR imaging camera.(Li = liver, Lu = lungs, H = heart, Tu. = tumor, S = spleen, K = kidneys) C+D) Pharmacokinetic profile for concentration of irinotecan or SN‐38 from nanotubes in blood plasma up to 24 h post i.v injection, reported in µM and µg/mL respectively, alongside indications of theoretical maximum/initial concentration based on average volume of circulating blood in mouse based on body weight. Error bars for all data represents the standard deviation of the mean from at least 3 technical replicates.

To monitor the in vivo behavior of the nanotubes, the synthetic protocol was modified to incorporate the NIR fluorophore AlexaFluor 647 into the structures (see Experimental Section). Fluorescent nanotubes were intravenously injected in subcutaneous CT26 xenograft‐bearing Balb/c mice. Three and 24 h after injection, mice were sacrificed, and *ex vivo* imaging was conducted to assess the biodistribution of the particles in various organs and tissues. The results indicated that accumulation primarily occurs in the liver and kidneys, as anticipated due to the natural opsonization and renal filtration processes (**Figure** **5**A,B).^[^ ^22^, ^23^ ^]^ The evidence of renal clearance via accumulation in the kidneys also suggests gradual disassembly of the nanotubes into their unimeric conjugates which are below the ultrafiltration size cut‐off. The variability observed in liver and spleen accumulation, as reflected by the standard deviations was noted, and likely due to inherent biological variability arising from differences in metabolism, clearance mechanisms, and organ blood flow between animals. Furthermore, there was a significant accumulation in the lungs, potentially attributable to mechanisms involving macrophages engulfing foreign particles present in the bloodstream for prolonged periods, offering an interesting avenue for further applications in lung disease therapeutics. Additionally, some evidence suggested nanotube accumulation within the tumor tissue at 3 h, slightly increasing after 24 h. Although the degree of tumor accumulation is low, the elongated nanoparticle can be retained within the tumor microenvironment, with sustained release of the drug, thus enabling the use of smaller doses when compared to small molecules.^[^ ^24^ ^]^

A pharmacokinetic profile of SN‐38 from nanotubes compared to irinotecan was established following intravenous administrations and collection of blood samples for analysis by LC‐MS (Figure 5C,D). Free SN‐38 was not tested in vivo due to poor aqueous solubility and a known unfavorable PK profile with rapid clearance.^[^ ^8^ ^]^ In line with expectations,^[25]^ the plasma irinotecan concentration decreased dramatically within the first 6 h of administration, whereas SN‐38 concentrations were markedly more sustained over the course of 24 h. A simple non‐compartmental analysis model was applied to reveal that the average half‐life of SN‐38 (**Table** **1**) was extended by almost 70% compared to irinotecan, with estimated total drug exposure (AUC) reaching over 6‐fold improvement within the 24 h timeframe. It is worth noting that the concentration of free/hydrolyzed SN‐38 was below the limit of detection, and the results reflect the SN‐38 still intact in prodrug nanotube form, demonstrating the relative stabilization of the drug in blood, which is anticipated to reduce immediate off‐target toxicity. This comparison highlights the value of the nanotubes in offering a substantially improved SN‐38 plasma exposure compared to the clinical drug candidate, tumor accumulation and an improved anti‐proliferative effect, particularly considering the higher potency of SN‐38.

**Table 1:** Calculated pharmacokinetic parameters comparing irinotecan versus SN‐38 from nanotubes using a non‐compartmental model.

	Irinotecan	SN‐38 from Nanotubes^a)^
**C _max_ [µM]**	29.6	48.2
**AUC 0‐t_24h_ [µM., 10** **hr]**	71.8	487.7
**T _1/2_ [h]**	15.8	26.7

^a)^ SN‐38 refers to the total i.e both prodrug and hydrolysed forms combined.

#### 2.4.2 Anti‐Tumor Efficacy

**Figure 6:**
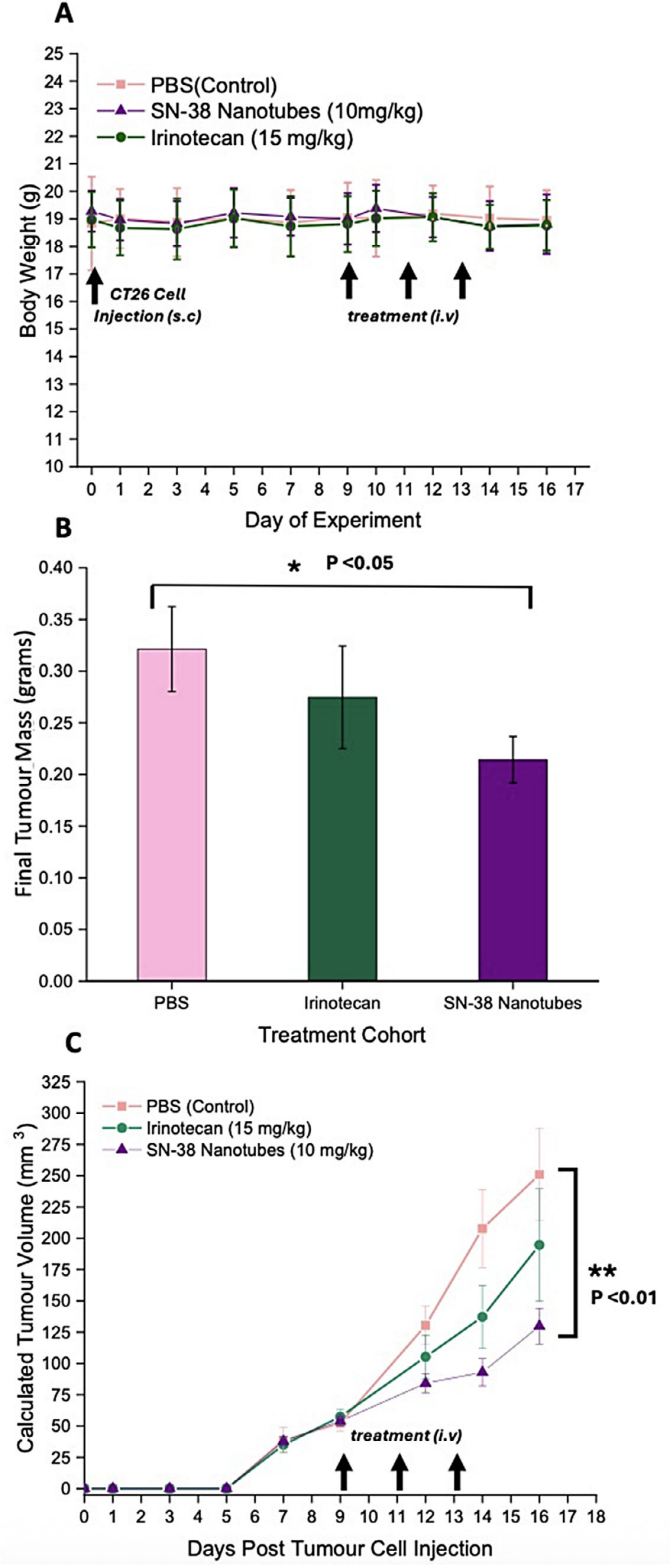
A) Anti‐tumor efficacy of treatments upon growth of CT26 xenograft model in Balb/C mice (F) as determined by physical caliper measurement following intravenous dosing in three repetitions. B) Final weight of excised tumor tissues on day 17 post s.c tumor cell inoculation. C) Body weight of mice cohorts throughout the experiment timeline. Error bars represent the standard error of the mean. (n = minimum of 8 for each cohort). P value calculated by Ordinary One‐Way ANOVA analysis in GraphPad Prism.

Since the SN‐38 nanotubes exhibit significantly prolonged plasma retention time, an anti‐tumor study was conducted on the CT26 Xenograft model in BALB/c mice, comparing them against irinotecan, and PBS control treatments. After establishing 50 mm^3^ tumors, mice were randomly assigned to three cohorts and administered every other day for a total of three doses (10 mg kg^−1^ SN‐38 and 15 mg kg^−1^ irinotecan, corresponding to approximately the same number of drug molecules introduced per injection). Importantly, both the SN‐38 nanotube treatment and irinotecan were well tolerated as evidenced by the maintenance of healthy body weight and consistent zero scoring for welfare check criteria in each mouse (**Figure** **6**C). Additionally, monitoring of tumor size (Figure 6A) revealed that both treatments exhibited some ability to inhibit tumor growth, with a significant difference observed between SN‐38 nanotubes and the PBS control (average tumor volume on day 16 post tumor cell inoculation: PBS (251 ± 37 mm^3^), Irinotecan (195 ± 45 mm^3^), SN‐38 Nanotubes (130 ± 14 mm^3^)). This effect was further validated by the weight of excised tumors on day 17, which showed that the SN‐38 treated cohort had limited the average mass (grams) by one‐third compared to the control group (Figure 6B). This marks the first in vivo evidence on tumor bearing animals to date of the potential of cyclic‐peptide nanotubes as delivery vectors capable of improving upon the outcomes of existing therapeutic agents. Furthermore, despite the limited nanotube tumor accumulation indicated by the biodistribution data, the successful anti‐tumor effect suggests an advantageous mechanism, possibly due to the much more favorable pharmacokinetic profile that might suggest the drug was delivered to the tumor cells in a sustained therapeutic concentration.

## 3 Conclusion

From this study, SN‐38 peptide‐polymer conjugate nanotubes emerge as a promising solution for overcoming the limitations of potent chemotherapeutic drugs with unfavorable in vivo properties. Through precise synthesis methods, these nanotubes achieved high drug loading efficiency and exhibited favorable self‐assembly properties, facilitating sustained drug release. In vitro experiments demonstrated significant cytotoxicity against colorectal cancer cells, outperforming irinotecan at levels like that of free SN‐38, which is not feasible to use in vivo. In fact, our pharmacokinetic and pharmacodynamic studies in mice showed that SN‐38 from nanotubes has a much more favorable profile with prolonged plasma retention and promising anti‐tumor efficacy superior to that of irinotecan. These findings highlight the potential of SN‐38 nanotubes as well‐tolerated therapeutic option for colorectal cancer, with implications for future clinical translation and improved patient outcomes.

## 4 Experimental Section

### Materials

7‐ethyl‐10‐hydroxycamptothecin (98%), irinotecan hydrochloride, and succinic anhydride were obtained from Sigma Aldrich (Merck). 4‐Acryloylmorpholine (98%) was obtained from Alfa Aesar. N‐Ethyldiisopropylamine (99%) and 2‐hydroxyethylacrylate (97%) were obtained from ACROS Organics. EDC.HCl was obtained from Carbosynth Ltd. PABTC and PABTC‐NHS were synthesized in‐house according to literature protocol.^25^ Alexa Fluor 647 amine dye, and all other solvents were obtained from Fisher Scientific.

### Synthesis of Cyclic Peptide Chain Transfer Agent

Cyclic peptide (1 eq) was dissolved in DMF alongside PABTC‐NHS (2 eq), to which DIPEA (3 eq) was added dropwise with stirring. The reaction solution was stirred for 3 h, following precipitation into ice‐cold diethyl ether three times to purify. The final pellet was dried under vacuum to afford a bright yellow solid.

### Synthesis of HEA‐SA Monomer

2‐hydroxyethylacrylate 1 eq), Succinic anhydride (5 eq), and DIPEA (10 eq) were dissolved in dichloromethane (100 mL) and stirred for 18 h before washing with DI water, 0.1 M HCl, and again with DI water before collection of the organic layer and drying over MgSO_4_. The solvent was removed by rotary evaporation to afford a thick brown oil.

### Synthesis of Cyclic Peptide Polymer Conjugate via Grafting From

The CP‐CTA was weighed into a vial, followed by the addition of DMSO. The vial was heated via heat gun to aid dissolution of the peptide at high temperatures, which was achievable but changed to a thick gel‐like consistency upon return to room temperature. The monomer and initiator were then added, and the vial shaken to allow even distribution of the contents, and a small sample taken for NMR analysis. Following this, the vial was charged with a magnetic stirrer bar and sealed before degassing with nitrogen line for 30 min. The vial was then placed into a heated oil bath with preset temperature (70 °C), allowing for the reaction to proceed for the specific time duration. Following completion of the first block, the second monomer was directly added to the solution alongside additional initiator and solvent, and the above repeated to instruct polymerization of the second block. The transition from thick gel to one of lower viscosity, then to a free moving solution was observed as the reaction proceeded. At the end of the reaction the solution was precipitated into ice‐cold ethyl acetate, followed by centrifugation to afford a solid pellet which was subsequently washed twice more with ethyl acetate before drying under vacuum to afford a pale yellow fluffy solid.

RAFT Polymerization Conditions were as Follows: [CTA]/[I] = 10, T °C = 70 °C, Time (hours) = Block 1 (HEA‐SA) – 18h+, Block 2 (NAM) – 6 h, [M]_o_ = 0.15 mol L^−1^ or less.

### Conjugation of SN‐38 to Cyclic Peptide Polymer Conjugate

CP‐polymer conjugate (1eq) was dissolved in DMF alongside SN‐38 (7‐ethyl‐10‐hydroxycamptothecin) (15 eq, 1.5 eq per an assumed 10 reactive sites) and DIPEA (30 eq). Next, EDC.HCL in DMF (20 eq) was added and the solution stirred overnight before precipitation into ice‐cold diethyl ether. The pellet was collected and redissolved in the minimum amount of dichloromethane, and this directly precipitated into ice‐cold methanol followed by centrifugation and pellet collection. This was repeated 2 times further, and the final pellet dried under vacuum to afford a light‐yellow solid.

### Fluorescent Labelling of Cyclic Peptide Polymer Conjugate

The procedure to fluorescently label the conjugates was identical to that described above, but with the inclusion of Alexa‐Fluor647 amine alongside the SN‐38 drug to achieve a dye labelling of 7% of all chains.

### Determination of Drug Loading Sample Weight Percentage

Full release of drug from the conjugate was achieved by dissolution of an accurately weight quantity (1–2 mg) in 1 mL PBS (pH 7.4) (5% EtOH) and adding 10 uL of 2 M NaOH. The sample was incubated at 37 °C overnight, before neutralization with 10 µL of 2 M HCl. The sample was then injected onto HPLC, and the drug content measured against a calibration curve to determine the loading.

### In Vitro Testing‐Drug Release Profile

A known quantity of final SN‐38 loaded peptide‐polymer conjugate (3‐4 mg) was weighed into a vial, to which 50 uL ethanol and 50 µL PBS (pH 7.4) was added. The vial was heated to aid dissolution, and diluted with PBS such that the ethanol content was less than 5% v/v. One hundred µL of the solution was transferred to multiple Eppendorf tubes either with or without mouse serum, closed, and transferred to a water bath (37 °C). At each time point, the Eppendorf's were transferred to ‐20 °C freezer and stored until the time of analysis. PBS only samples were directly injected onto HPLC for analysis. Mouse serum samples were precipitated with a 3‐fold volume of ice‐cold acetonitrile (5% TFA), centrifuged to separate proteins, and the supernatant injected onto HPLC for analysis. The extent of drug release at each time point was compared to a separate sample to which 5 µL of 2 M NaOH had been added to instruct total 100% hydrolysis of the drug, followed by neutralization with 5 µL of 2 M NaOH. The AUC signal in HPLC at wavelength 365 nm was used to determine the extent of drug hydrolysis.

### In Vitro Testing‐Cell Culture

CT26.WT cells were acquired from the American Type Culture Collection (ATCC 2638) and cultured in Dulbecco's Modified Eagle Medium (DMEM) supplemented with foetal bovine serum (FBS, 10% v/v) and 1% Penicillin/Streptomycin (v/v) in a CO2 (5%) incubator at 37 °C. Cells were typically grown to 70–80% confluence before passaging, and cells were not used past 20 passages total.

### In Vitro Testing‐Sample Preparation

SN‐38 and Irinotecan were accurately weight, dissolved in cell culture‐appropriate DMSO, and then diluted with PBS (pH 7.4) before filtration. The SN‐38 nanotubes were accurately weight, dissolved in EtOH/PBS, and diluted to the appropriate concentration with PBS before filtration through 0.2 µM Cellulose Acetate Filters. To ascertain the correct drug concentration post filtration (to account for sample loss), a small aliquot was taken and submitted to forced hydrolysis studies with HPLC analysis to confirm the final drug concentration. All PBS stock solutions were then diluted at least 10‐fold with cell media to afford <10% PBS and less than 1% EtOH or DMSO.

### In Vitro Testing‐MTS Cell Viability Assay

CT26 cells were seeded in a 96‐well plate at a density of 2000 cells per well and incubated for 24 h. Then, the media was replaced with 100 µL of the prepared drug or conjugate in media at a range of concentrations and incubated for a further 48 h. The wells were then treated with 20 µL of MTS reagent (Promega, CellTiter 96 AQ_euous_ One Solution Cell Proliferation Assay) for 4 h, before UV absorbance reading on Biotek Cytation 3 Plate reader (490 nm). The relative cell viability was determined by comparison to untreated cells or cells with DMSO or EtOH at a range of concentrations.

### In Vitro Testing‐Dead Cell Assay by Flow Cytometry

CT26 cells were seeded in a 24 well plate at a density of 50000 cells per well and incubated for 24 h. The wells were refreshed with media with the prepared drug or conjugate at a concentration of 2.5 µM and incubated for 48 h. The cells were washed with PBS, harvested via trypsin, resuspended in PBS and treated with the Membrane Permeability/Dead Cell Kit (YO‐PRO‐1 and PI, Invitrogen) before analysis on the BD Biosciences LSR II Flow Cytometer. Data analysis was performed with BD FACSDiva Software.

### In Vitro Testing‐Cell Cycle Assay by Flow Cytometry

Cell Cycle determination followed the same initial treatment protocol as above. After sample harvesting, cells were fixed with 70% ethanol on ice for 30–45 min, before washing thrice with PBS and direct resuspension in FxCycle PI/RNase Staining Solution (Molecular Probes, Life Technologies). Samples were incubated at room temperature, protected from light for 15–30 minutes before analysis via flow cytometry.

### In Vitro Testing‐Cell Viability Assay of 3D Spheroids

CT26 cells were harvested and seeded in a non‐tissue culture‐treated, U‐bottom‐shaped 96‐well plates at a density of 500 cells per well (200 µL media per well), before centrifugation at 500 xG for 10 minutes. The plate incubated and the spheroids allowed to form over a period of days (typically 5–8). Upon formation of spheroids, 100 µL of media in each well was replaced with 100 µL of drug or conjugate at a concentration of 20 µM to give final concentration of 10 µM. Non‐treated/Control spheroids received fresh media. The plates were incubated for 5 days with brightfield microscope images taken before and after to compare size.

### In Vivo Testing

All experiments involving live animals was conducted in accordance with the University of Warwick's AWERB and United Kingdom Home Office Animal Scientific Procedures Act (1986). The specific work conducted was approved under Project License Number PP3644080. Female BALB/cAnNCrl mice (6‐8 weeks) were purchased from Charles River Laboratories UK and allowed to acclimatise for at least 7 days prior to any of the experiments described below.

### Generation of CT26 Xenograft Mice

One day prior to tumor cell inoculation, the right flank/back of each mouse was shaved. CT26 cells were grown in Dulbecco's Modified Eagle Medium (DMEM) (+10% Foetal Bovine Serum, 1% Penicillin/Streptomycin), harvested via trypsinization, and resuspended in DMEM without additives to a concentration of 10 million cells per mL. Mice were placed under anaesthesia (2% isoflurane) and 100 µL of the suspension (1 million cells) injected subcutaneously into the right flank (briefly sterilized with ethanol wipe) with a 29G insulin needle. Mice were transferred to a clean cage with supplementary apple sauce until full recovery. The body weight was recorded at least 3 times a week, and the tumor site palpated until growth could be measured by digital callipers. The volume was calculated using the following equation: Tumor Volume (mm^3^) = (shortest diameter)^[^ ^2^ ^]^ * longest diameter * 0.5

### Biodistribution

The tumor bearing mice were intravenously injected with 100 µL of Alexa Fluor 647 labelled SN‐38 nanotubes (10 mg kg^−1^) and subsequently provided with apple sauce to aid recovery. At specific time points (3 h, 24 h), the mice were culled via schedule 1 cervical dislocation method, and the organs/tissues extracted before imaging with the Biospace Photonimager (Biospace Lab, France) followed by image analysis with M3Vision Software (Biospace Lab, France). Acquisition Mode: FLI, Integration (1000 ms per frame, 5 frames), Excitation 637 nm, Background 587 nm, Emission 697 nm, Filter cut off = 680–715 nm (band pass).

### Tumor Efficacy Study

Treatment of thirty tumor bearing mice was initiated when the average tumor volume of each mouse/group reached 50 mm^3.^ Mice were randomly assigned to three groups (n = 10) and injected intravenously with 50 µL of the following treatments on every other day up to a maximum of three doses – PBS (control), SN‐38 loaded nanotubes (10 mg kg^−1^ drug) in PBS, or Irinotecan (15 mg kg^−1^) in PBS. The mice were monitored (body weight, tumor volume) at least thrice weekly until 16 days after tumor implantation. However, three mice were culled early due to tumor ulceration or migration. The remaining mice were culled on day 16 post implantation, where the threshold for euthanasia due to excessive tumor burden (12.5 mm in any single direction) was close to being met for many of the cohort, in accordance with the project license PP3644080. Tumor tissues were then excised and weighed (Supporting Information).

### Pharmacokinetics Study

Non‐tumor bearing mice were injected intravenously with either PBS (pH 7.4, 100 µL), SN‐38 loaded nanotubes (10 mg kg^−1^ drug, PBS carrier, 100 µL), or Irinotecan (15 mg kg^−1^, PBS carrier, 100 µL). At indicated time points (5 min, 90 min 3 h, 6 h, 24 h) the mice were cervically dislocated, and upon confirmation of death, blood was collected from the thoracic cavity. The volume of blood was diluted 3‐fold with PBS (pH 7.4) and then stored at −20 °C until time of analysis. Prior to analysis, solutions were defrosted, and then a known volume added to an Eppendorf with known volume of ice‐cold acetonitrile to precipitate out proteins. The Eppendorf was centrifuged, and the supernatant collected, filtered through 0.2 µM PTFE filter, and transferred to HPLC vials for analysis via LC‐MS. The signal from each sample was compared to that of a standard calibration curve to determine the plasma drug concentration. Subsequently, data was analyzed with the non‐compartmental model (NCA) to calculate some specific PK parameters using the open‐access application at https://dash.gallery/dash‐pk‐calc/.

## Conflict of Interest

The authors declare no conflict of interest.

## Author Contributions

S.K.H. contributed to writing (review and editing, original draft), visualization, validation, methodology, investigation, data acquisition and experimental work, formal analysis, and conceptualization. S.P. contributed to writing (review and editing, original draft), visualization, validation, supervision, software, resources, project administration, methodology, investigation, funding acquisition, formal analysis, data curation, and conceptualization. M.Z. (TEM imaging) contributed to investigation and data acquisition/experimental work. R.D. (in vivo studies) contributed to resources, methodology and experimental setup/planning/work, and writing (editing). S.K.R. (in vivo studies) contributed to methodology and experimental setup/planning.

## Supporting information



Supporting Information

## Data Availability

The data that support the findings of this study are available from the corresponding author upon reasonable request.
