# Supplementary material for: Cyclic Peptide–Polymer Conjugate Nanotubes for Delivery of SN‐38 in Treatment of Colorectal Cancer Model
Source: Adv Healthc Mater. 2025 Oct 10;15(4):e02527. doi: 10.1002/adhm.202502527 (PMC12836451; doi:10.1002/adhm.202502527)
Supplement: Supplementary file 1 — Supporting Information [file ADHM-15-0-s001.pdf]

ADVANCED HEALTHCARE MATERIALS

Supporting Information

for *Adv. Healthcare Mater.*, DOI 10.1002/adhm.202502527

Cyclic Peptide–Polymer Conjugate Nanotubes for Delivery of SN-38 in Treatment of Colorectal Cancer Model

*Sophie K. Hill, Min Zeng, Santhosh Kalash Rajendrakumar, Robert Dallmann and Sébastien Perrier**

Supporting Information

Cyclic Peptide – Polymer Conjugate Nanotubes for Delivery of SN-38 in Treatment of Colorectal Cancer Model

Sophie K. Hill [a, b], *Min Zeng* [a] ,*Santhosh Kalash Rajendrakumar* [a] ,

Robert Dallmann [b,c], *Sébastien Perrier* [a,b,c*]

[a] Department of Chemistry, University of Warwick, Gibbet Hill Road, Coventry CV4 7AL, UK.

[b] Warwick Medical School, University of Warwick, Coventry, CV4 7AL, UK.

c Zeeman Institute for Systems Biology and Infectious Disease Epidemiology Research, University of Warwick, Coventry, CV4 7AL, UK

[c] Faculty of Pharmacy and Pharmaceutical Sciences, Monash University, 381 Royal Parade, Parkville, VIC 3052, Australia.

** Corresponding Author: S.Perrier@warwick.ac.uk*

Materials

Amino Acids Fmoc-D-Leu-OH, Fmoc-L-Lys (Boc)-OH, Fmoc-L-Trp(Boc)-OH were obtained from Sigma Aldrich (Merck). HCTU was obtained from Novabiochem. Piperidine (99%) and Trifluoroacetic Acid (99%) were obtained from Thermo Scientific. DMTMM.BF_4 was synthesised in house according to literature protocol.[2] All other solvents were obtained from Fisher Scientific.

Characterisation

Characterisation – Mass Spectrometry. Low resolution ESI-MS was performed on the Agilent 6130B Single Quad ESI Instrumentation. Samples were analysed in both +ve and-ve mode, in a solvent of methanol.

Characterisation – High Performance Liquid Chromatography (HPLC). High performance liquid chromatography was performed on a Shimadzu Prominence Instrument equipped with photo diode array detector (PDA). The column installed was the Agilent Technologies Poroshell : 2.7 µm, 4.6 x 150mm,C18. Water (HPLC grade + 0.04% TFA) and Acetonitrile (HPLC grade + 0.04% TFA) were implemented as the A and B phase eluents respectively and chromatography was performed using a gradient of B 10% to 95% over 25 minutes.

Characterisation – Liquid Chromatography – Mass Spectrometry (LC-MS)

LC-MS was performed on instrumentation equipped with the Agilent 1260 Infinity Range Pumps, Autosampler, UV detector, Column Oven

Characterisation – Nuclear Magnetic Resonance Spectroscopy (NMR). ^{1}H NMR Spectra were obtained using a Bruker DPX – 300 MHz or DPX – 400 MHz spectrometer and deuterated solvent peaks were used as internal reference. Shift values (δ) are reported in units of ppm. Characterisation of compounds was performed in deuterated dimethyl sulfoxide (DMSO - $^{\delta}$H – 2.5 ppm). Each spectra was baseline corrected and analysed using ACD Labs ^{1}D NMR software.

Characterisation – Size – Exclusion Chromatography (SEC) / Gel Permeation Chromatography (GPC).. GPC DMF data was obtained on the Agilent 1260 Infinity II instrument with DMF + 5 mM NH4BF4 as the eluent and differential refractive index detection. The instrument was run with two PolarGel H columns (300 mm x 7.5 mm) and a PolarGel 5uM guard column at 50 °C and a flow rate of 1 mL min^{-1} Samples were prepared in a suitable eluent solvent to at least 1 mg/mL concentration and filtered through polytetrafluoroethylene (PTFE) membranes with 0.2 µM pore size. The instrument was calibrated with poly (methyl methacrylate) standards. Data analysis of output results was performed using the Agilent GPC/SEC software.

Characterisation – Small Angle X-Ray Scattering (SAXS). SAXS samples were submitted to the Warwick University Scientific Services (WSS) X-Ray Diffraction Facility. Samples were analysed using the 5m Xenocs Xeuss 2.0 SAXS instrumentation equipped with Cu microfocus source (λ =1.54189 Å)collimated with scatterless slits. The beam diameter was 0.8 mm and the scattering was measured by a Pilatus 300k detector (pixel 0.172mm x 0.172mm). The distance between the measured sample and detector was calibrated using silver behenate. Samples were dissolved in PBS buffer and placed in capillary vessel prior to measurement, which typically took place at 25o C for 4h. To rescale data and absolute intensity, glassy carbon was utilised as a standard. Measured Data was extracted and analysed using SasView software. SLD's for the peptide core, drug molecule and polymer corona were calculated as follows and used as fixed parameters. The scattering length density (SLD) can be described as the scattering intensity of a specific analyte and can be defined as the total of scattering lengths, bi of for each N number of atoms within a specified particle volume, Vm. For diblock copolymers the SLD was calculated based on the mole fraction of each monomer present in the overall polymer.

$$SLD = \frac{\sum_{i=1}^{N} b_i}{Vm}$$

The values used for atomic scattering lengths and accurate atomic weights were those specified in the NIST database.

Characterisation – Transmission Electron Microscopy

TEM images were recorded on a 200 kV JEOL PLUS transmission electron microscope. Samples were prepared by dropping 10 μL of 1mg/mL sample solution onto copper grid, with the droplet being removed by filter paper after 60 s. The process was repeated three times before staining from the vapor of 0.5 wt% ruthenium tetroxide (RuO_4) solution, and subsequently allowing the grid to dry at room temperature before imaging.

Synthetic Protocol

Peptide Synthesis

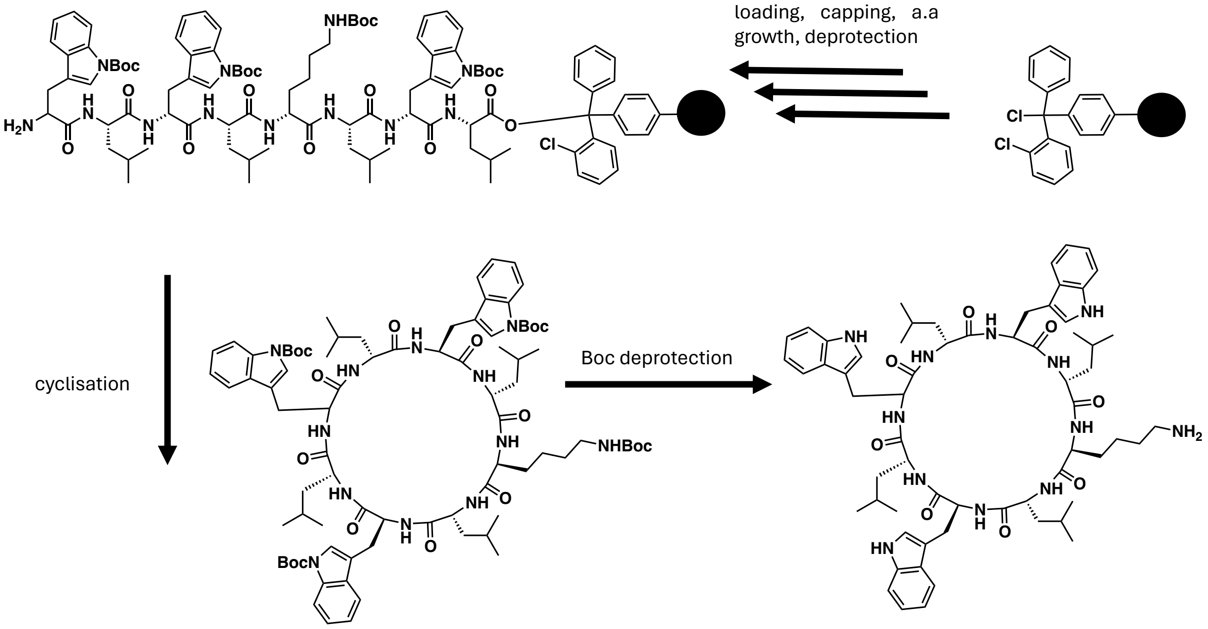

Linear Peptide Synthesis

Peptide was produced either by the Prelude Peptide Synthesiser, or manually in a sintered syringe. 2-chlorotrityl chloride resin (1.1 mmol g-1) was measured and swollen in dichloromethane for 30 minutes before being drained. Next, the first amino acid was loaded onto the resin by addition of Fmoc-D-Leu-OH (200 mM in DMF) and DIPEA (400 mM in DMF) and mixing for 2 h. The solutions were drained and unreacted resin sites were capped with DCM/MeOH/DIPEA (85/15/5 volume ratio) for X hours. The solution was drained and washed with dichloromethane (x3) and dimethylformamide (x3) followed by deprotection of the Fmoc group with piperidine for 5 minutes (20% in DMF). The deprotection stage was repeated twice to ensure completion and the resin was again washed with DCM and DMF. Coupling of the next amino acid was achieved by addition of Fmoc-L-Trp(Boc)-OH and HCTU/NMM and mixing for 30 minutes. The solution was replaced with fresh reagent and the coupling

allowed to proceed for a further 30 minutes, followed by washing of the resin with DCM and DMF. The Fmoc deprotection and a.a coupling stages were then repeated until all the amino acids in the sequence had been added. Finally, the last Fmoc protecting group was removed with piperidine solution, and the peptide was cleaved from the resin using 20% HFIP in DCM solution followed by washings with DCM to collect the free linear peptide. The solvent solution was removed in vacuo to afford the linear peptide as a yellow solid.

Cyclisation: Linear peptide (1 eq) was dissolved in a large excess of dimethylformamide (150 mL), to which a solution of DMTMM.BF_4 (1.4 eq) in DMF (20 mL) was added dropwise over a 30 minute period. The solution was then stirred for 4 days and upon completion of cyclisation (as determined by ESI-MS) the majority (90%) of solvent was removed in vacuo to afford a thick clear/white sludge. Approximately 50 mL of an ice cold 1:1 MeOH/Water solution was added, the flask shaken and the contents then transferred to two 50 mL falcon tubes before centrifugation at 9000g for 10 minutes to afford a white pellet. The pellet was washed twice more with MeOH: Water and the final pellet dried under vacuum to afford a white/beige solid.

Deprotection : Boc removal was achieved via the addition of a 95:2.5:2.5 solution of TFA:TIPS:Water solution added to a known quantity of the cyclic peptide, with stirring for 2 hours. Following confirmation of deprotection by ESI-MS the TFA was removed via evaporation under a light stream of nitrogen, and the concentrated solution precipitated into ice cold diethyl ether before centrifugation to afford a pellet. The pellet was dried under vacuum to afford a beige solid.

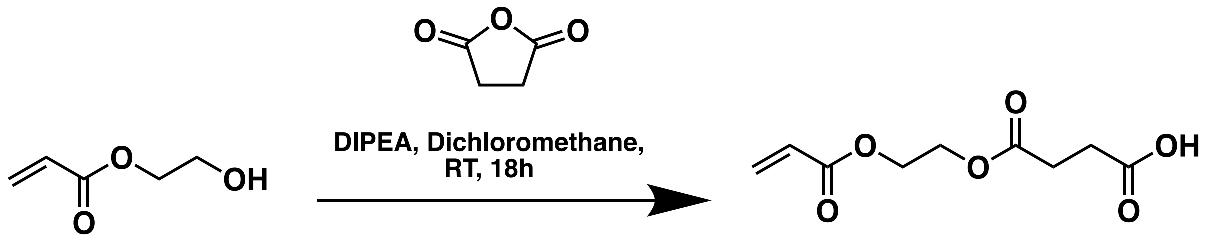

Scheme S1: Synthetic scheme of the monomer linker 2-(acryloyloxy)ethyl succinate.

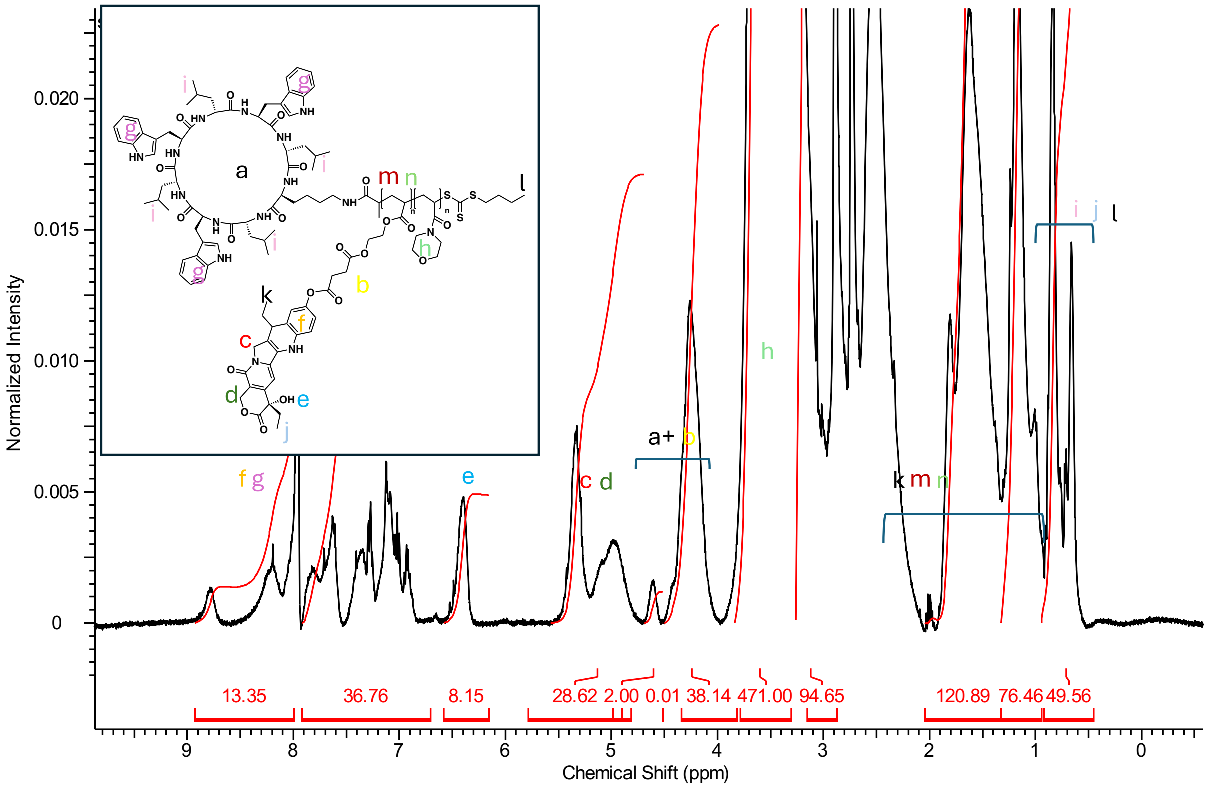

Figure S1: ^{1}H NMR spectrum of the drug loaded conjugate.

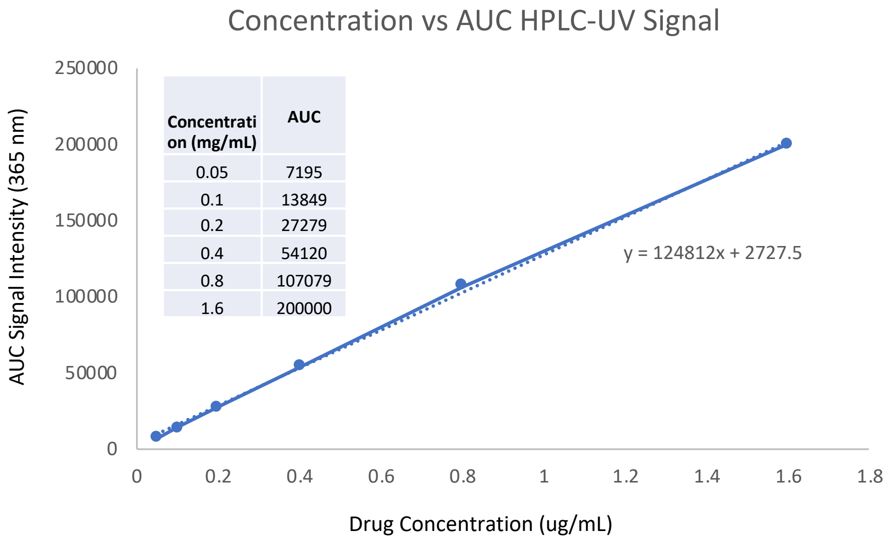

Figure S2: Calibration curve for SN-38 release determined by HPLC-UV detector.

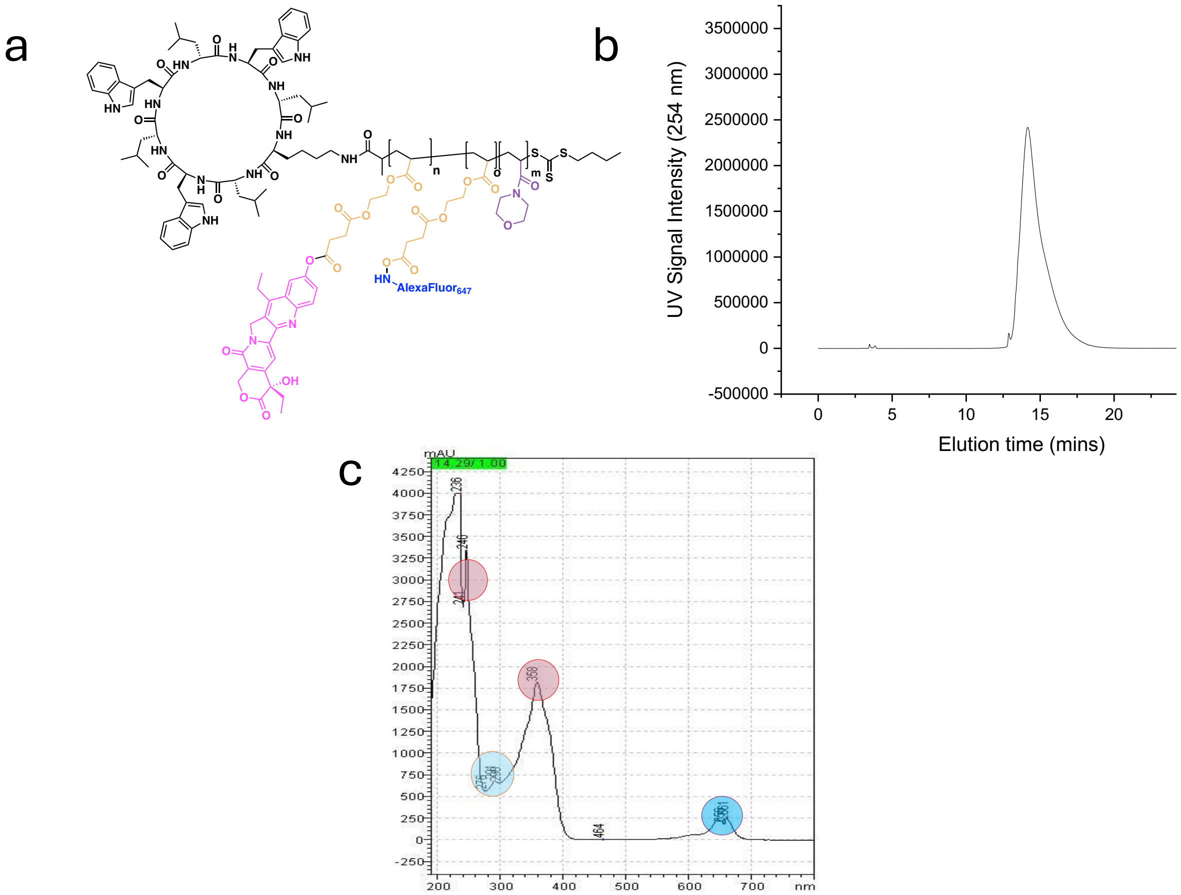

Figure S3: a) Structure of the AlexaFluor647 labelled SN-38 loaded CPNT, b) HPLC Chromatogram of the Purified Conjugate without free drug or dye, c) UV Absorbance of the conjugate with requisite peaks for peptide tryptophan, SN-38 drug and AlexaFluor647 dye.

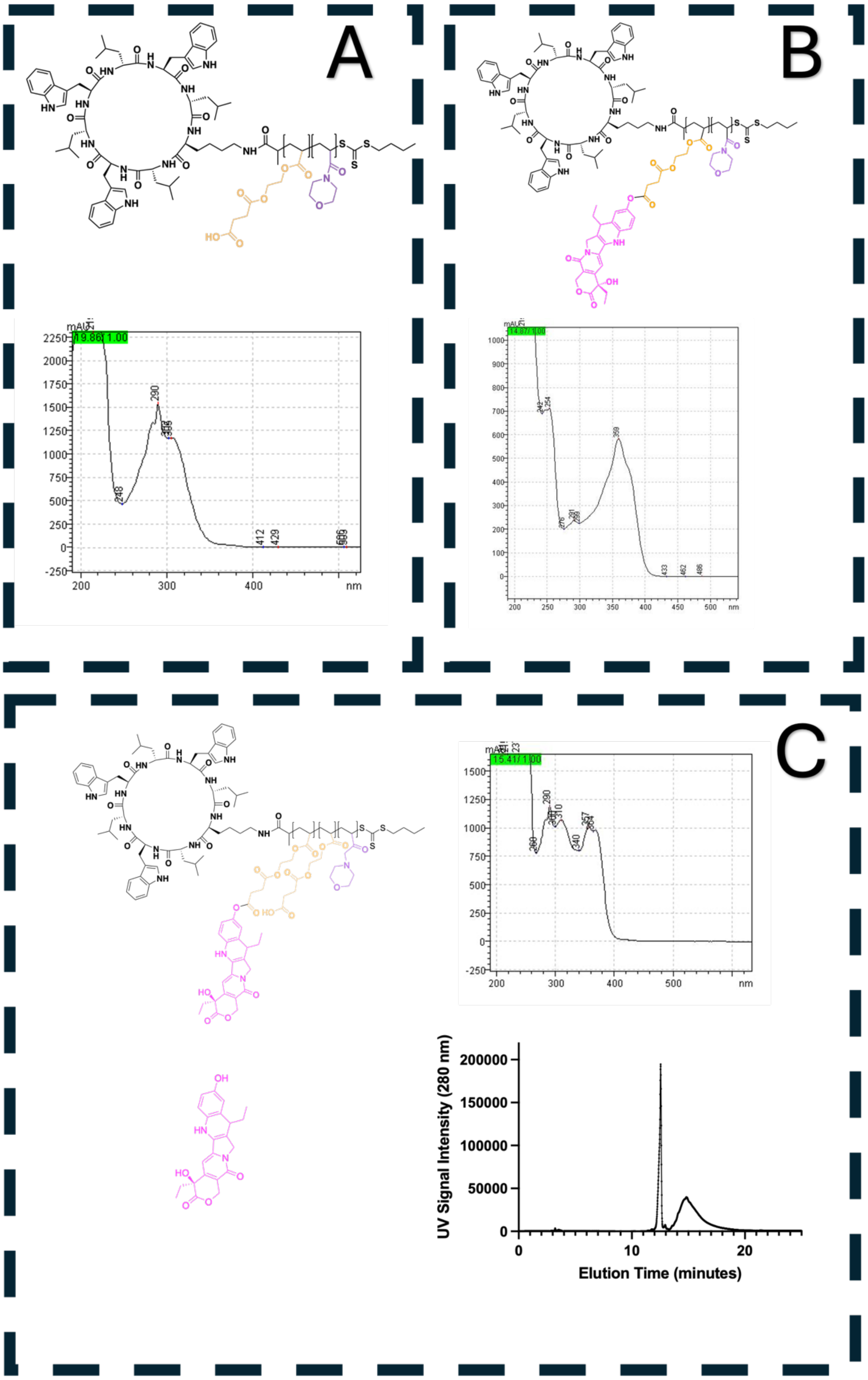

Figure S4: HPLC chromatogram of the release of SN-38 after 72h in mouse serum.
A) Chemical structure of non-drug loaded material + UV spectrum showing peaks at 280 nm (peptide tryptophan) and 309 nm (polymer trithiocarbonate); B) Chemical structure of drug loaded material + UV spectrum showing strong SN-38 peaks at 254 nm and 365 nm overlapping with the peak at 280/309 nm of the peptide/polymer conjugate; C) Chemical structure of proposed major degradation products

following 72h incubation in mouse serum (partially hydrolysed drug-peptide-polymer conjugate, and freely released SN-38). HPLC profile after 72h incubation in mouse serum (following solvent extraction) showing 2 major species (sharp peak 12 mins SN-38, broad peak 12-18 minutes = drug loaded peptide-polymer conjugate (multiple degrees of drug conjugation per unimer due to partial hydrolysis). The UV spectrum shows reduction of the SN-38 PEAK (357nm) as a result of drug hydrolysis/release, and a strong retention of the 309 nm trithiocarbonate peak, suggesting that aminolysis of trithiocarbonate end group is negligible and not a likely degradation product under biological conditions.

References

1. C. J. Ferguson, R. J. Hughes, B. T. T. Pham, B. S. Hawkett, R. G. Gilbert, A. K. Serelis and C. H. Such, Effective ab Initio Emulsion Polymerization under RAFT Control, *Macromolecules*, 2002, **35**, 9243–9245.
2 . S. A. Raw, An improved process for the synthesis of DMTMM-based coupling reagents, *Tetrahedron Lett.*, 2009, **50**, 946–948.
